# Equine Activities Influence Horses’ Responses to Different Stimuli: Could This Have an Impact on Equine Welfare?

**DOI:** 10.3390/ani9060290

**Published:** 2019-05-29

**Authors:** Tiago Mendonça, Cécile Bienboire-Frosini, Izabela Kowalczyk, Julien Leclercq, Sana Arroub, Patrick Pageat

**Affiliations:** 1 Behavioral and Physiological Mechanisms of Adaptation Department, Research Institute in Semiochemistry and Applied Ethology (IRSEA), 84400 Apt, France; c.frosini@group-irsea.com; 2 Animal Experimentation Department, IRSEA, 84400 Apt, France; izabela.kowalczyk@cea.fr (I.K.); j.leclercq@group-irsea.com (J.L.); 3 Statistical Analysis Department, IRSEA, 84400 Apt, France; s.arroub@group-irsea.com; 4 Semiochemicals’ Identification and Analogs’ Design Department, IRSEA, 84400 Apt, France; p.pageat@group-irsea.com

**Keywords:** behavior, horse, equitation, heart rate variability, reactivity, welfare

## Abstract

**Simple Summary:**

Horses are required to perform a wide variety of activities. Training a horse for these activities may influence the horse’s perception of and reactions to different stimuli. This study investigated the reactivity and emotional responses of horses involved by humans in different equine activities (dressage, jumping, eventing and equine-assisted activity/therapy) by studying their physiological and behavioral responses to different stimuli. A test setting with five phases was created to test equine responses to five different stimuli and compare these responses among horses from the different disciplines. It was demonstrated that the horses involved in the different activities had different responses, both physiologically and behaviorally, to the studied stimuli. These findings suggest that training a horse for a specific activity modifies the perception of stimuli and the strategies that the horse uses to balance its emotional state. Thus, horses involved in different activities probably behave differently according to their training. Such information is of great importance in improving training methods, with the aim of increasing equine welfare.

**Abstract:**

The learning and cognitive challenges that horses may face differ according to the activities in which they are involved. The aim of this investigation was to study the influence of equine activities on the behavioral responses and autonomic nervous system (ANS) activity of adult horses. Forty-one horses were divided into four groups: dressage (9), jumping (10), eventing (13) and equine-assisted activity/therapy (9). A test was created to compare the horses’ behavioral and physiological responses to different stimuli. The goal was always to obtain a treat. To study the ANS activity, heart rate variability was assessed using the standard deviation of the R-R intervals (SDNN), square root of the mean of the sum of the squares of differences between successive interbeat-intervals (RMSSD) and low frequency/high frequency (LF/HF). To assess behavioral responses, video analysis was performed considering the following behaviors: exploration, interactions with another horse, and latency to approach. Significant differences in SDNN (DF = 3; F = 3.36; *p* = 0.0202), RMSSD (DF = 3; F = 4.09; *p* = 0.0078), LF/HF (DF = 3; F = 4.79; *p* = 0.0031), exploration (DF = 3; F = 5.79; *p* = 0.0013) and latency to approach (DF = 3; F = 8.97; *p* < 0.0001) were found among horses from different equine activities. The activity that adult horses practice appears to influence behavioral and physiological responses to different stimuli, thus impacting equine welfare.

## 1. Introduction

The concept of animal welfare includes both physical and mental elements, suggesting that emotions are an important component of welfare [1,2]. Animal emotions have been widely studied and many studies have investigated the influence of emotions on animal welfare [3,4,5]. Stress has often been associated with negative emotions [6], and the latter has been associated with poor animal welfare [1].

Some authors have suggested that horses from different activities might express their emotions through different behaviors [7,8]. For example, fear reactions differ between horses from different activities [9]. Jumping horses appear to be less reactive to frightening stimuli than dressage horses [9]. The cited study suggested that jumping horses may have a genetic component involved in the development of such behavior, while no correlation was found between genetics and the development of behavior in dressage horses. Learning not to respond to frightening stimuli may be associated with a habituation or desensitization to these stimuli through training, but horses might not generalize this reduced fear response to all potentially fear-inducing situations related to competition and training [10].

Learning capacities are influenced by emotions [3], which means that horses trained to perform different equine activities may use different behavioral and physiological strategies to adapt to the training conditions in which they are involved. These differences could have consequences in the development of the animals’ behavioral traits and emotional management [11].

The learning and cognitive challenges that horses may face differ with the sports discipline they practice [12]. Dressage horses must discriminate between similar cues provided by riders, while jumping horses must learn to jump obstacles, while ignoring the obstacle color or structure [12]. Eventing horses must combine the needs of dressage and jumping horses [12]. Equine-assisted activity/therapy (EAA/T) involves activities that include both recreational and therapeutic interventions for people with mental and/or physical conditions [13,14,15]. Horses involved in these activities may experience stress induced by the inconsistency of the patients’ contact or cues, as these people are usually experiencing physical, mental or combined medical conditions [16,17].

As a social species, horses devote a significant amount of their time to intra- and interspecific interactions, and even to reconciliation after conflict [18,19]. The husbandry conditions and management of these animals should encourage socialization because equine behavioral and emotional responses might be influenced by the animals’ sociability [20]. Housing conditions that allow horses to interact with conspecifics have been associated with an improvement in learning abilities and training performance [21]. Additionally, housing conditions that involve social isolation have been associated to increases in psychological stress expressed by behavioral and physiological changes [22,23]. These findings suggest that improving the welfare (e.g., improving housing conditions) of horses may improve training performance as well. Nevertheless, training performance could also be influenced by other factors [8].

Stress-related responses have been associated with an increase in the activity of the hypothalamic-pituitary-adrenocortical (HPA) axis, adrenomedullary system and autonomic nervous system (ANS) in horses [24]. Some studies have investigated the HPA axis through the assessment of serum or salivary cortisol [15,25,26]. Other studies have investigated the ANS through the assessment of heart rate variability (HRV), which is a noninvasive technique for studying the ANS responses of horses [27]. Additionally, HRV analysis in horses has often been used to study emotional responses to cognitive [3] and temperament tests [11], and as an indicator for equine welfare assessments [27].

Sympathetic activity may be modified by psychological states without any perceived alteration in heart rate (HR) or in respiratory rate [28]. However, modifications in sympathetic activity should be reflected in HRV [27]. HRV might be useful to study sympathetic and parasympathetic activities during psychological or physical challenges via parameters such as the low frequency/high frequency (LF/HF) ratio [27].

HRV outputs include time domain analysis and frequency domain analysis. Time domain analysis has different parameters [27,29]. The most commonly used parameters to study HRV in horses are the HR, the standard deviation of all the R-R wave intervals (SDNN) and the square root of the mean of the sum of the squares of differences between successive interbeat-intervals (RMSSD) [29,30]. Frequency domain analysis includes parameters that are influenced by the respiratory rate, which varies according to species; therefore, the frequency band widths should be adapted in this type of analysis [31,32]. These band widths concern the following parameters: LF and HF. However, there is no established agreement concerning these frequency band widths. Some authors suggest that, for LH and HF, the band width thresholds should be 0.01–0.07 Hz and 0.07–0.6 Hz, respectively [33,34], while other authors suggest thresholds of 0.005–0.07 Hz for LH and 0.07–0.5 Hz for HF [32,35].

Each of the described parameters reflects different aspects of HRV [29]. The HR represents the net interactions between vagal and sympathetic regulation [27]. The SDNN reflects the long-term variability of cardiac activity and could be influenced by both the sympathetic and parasympathetic systems of the ANS [27,29]. The RMSSD represents high-frequency beat-to-beat variations reflecting the vagal activity [27,29]. Notably, no agreement has been established regarding the meaning of LF. Some authors have suggested that this parameter may represent sympathetic activity [27,36], while others have suggested that LF may represent both sympathetic and parasympathetic activities [32,35]. Additionally, some have suggested that the sympathetic tone should not be directly derived from HRV parameters [27,33]. In contrast, HF is commonly considered a marker of the cardiac vagal tone and, consequently, the parasympathetic system [27,29,33]. Finally, the most widely accepted aspect of frequency domain analysis is that the LF/HF ratio parameter reflects the sympatho-vagal balance or the sympathetic-parasympathetic balance [27,29,37].

The noninvasive character of and the information provided by HRV make this assessment an interesting tool for studying ANS activity in horses during physical or psychological challenges [14].

Therefore, the aim of the present investigation was to study the influence of the activity in which animals were involved on the behavioral responses and ANS activity of adult horses. We hypothesized that the behavioral responses and the ANS activity of horses were influenced by the activity in which horses were involved.

## 2. Materials and Methods

This investigation included two experiments. The first experiment investigated equine behavior and physiological responses under different types of stimuli. The second experiment investigated the influence of several equine activities on horses’ behavior and physiological responses.

This project was approved by IRSEA’s (Research Institute in Semiochemistry and Applied Ethology) Ethics Committee (C2EA125) and the French Ministry of Research (APAFIS Process number 11949).

### 2.1. Population

The horse populations differed between the two experiments. Common inclusion criteria were as follows: horses should be more than 4 years old, be performing any equestrian or therapeutic activity at the time of the experiment, and according to a physical consultation performed by a veterinary practitioner, should be healthy. All horses included in the experiments had access to paddocks during the daytime and to a single box during the night. On rainy days, the horses were kept indoors.

In the first experiment, the population of horses comprised forty-one horses (1 stallion, 23 geldings and 17 females) with a mean age of 10.41 ± 4.28 years old. These horses were involved in one of the following activities in their usual daily lives: jumping, eventing, dressage, endurance, reining or leisure. Leisure horses comprised horses that were involved in various riding activities (dressage, jumping, reining, equestrian rides) with no specific purpose, as a hobby of the rider.

For the second experiment, as part of the inclusion criteria, horses had to be involved in one of the following equine activities as part of their usual routine: jumping, eventing, dressage or EAA/T. These horses had been involved in one of these activities since the age of 3–4 years. To reduce the number of animals used for research proposals and to respect legislation concerning ethics and equine welfare, particularly the 3R’s rule, data obtained in the first experiment concerning horses involved in jumping, eventing and dressage were used for the second experiment. Nine new horses involved in EAA/T were also included. Ultimately, a population of forty-one horses (25 geldings and 16 females) with a mean age of 10.76 ± 3.99 years old was considered for this experiment. The population was divided into the following groups: dressage (9), jumping (10), eventing (13) and equine assisted activity/therapy (9).

### 2.2. Study Design and Data Collection

Each experiment was divided into different phases (described below). The first experiment investigated the phase effects to understand the behavioral and physiological responses of the tested horses in the different phases. The second experiment studied two factors: activity and phase. The phase factor was studied to ensure that any possible observed significant difference in activity was not influenced by a cross effect between phase and activity.

The setting and testing conditions were the same for both experiments.

**Figure 1:**
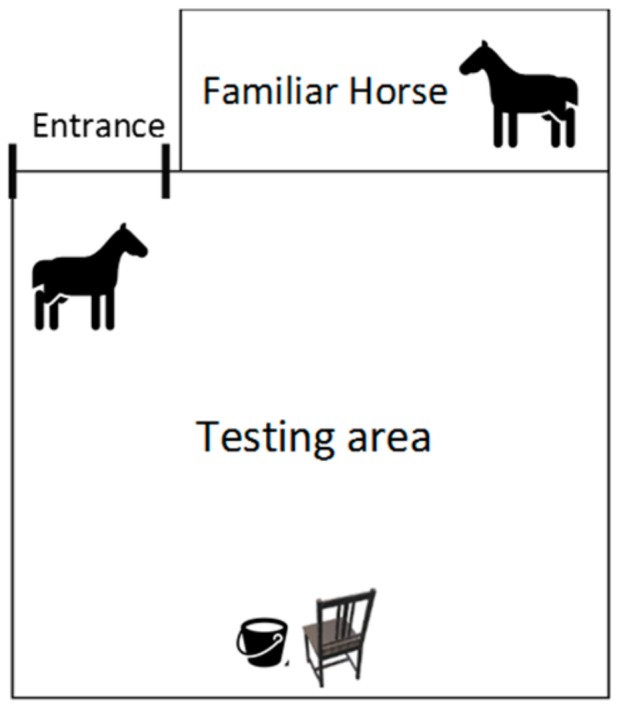
Scheme of the testing area. This setting was designed to test horse reactivity to different stimuli, while avoiding social isolation.

A testing area was created (Figure 1) to investigate equine physiological and behavioral responses to varying stimuli. The testing area was made of a 10 m^2^ paddock with an open field structure. A smaller paddock attached to the testing area hosted a familiar horse during the trials, respecting the horse’s need for interaction with other horses [21]. The familiar horses did not participate in the experiments and were provided with hay and water. A chair and a bucket containing treats (carrots, commonly used as treats in this equine population) were positioned at the side of the testing area opposite to the testing area’s entrance. The horse approaching and obtaining the treats from the bucket was always the goal.

A habituation period was used to let the horses spontaneously explore and get used to the setting and to create the motivation to approach the bucket and acquire the treats. This period lasted no more than 5 min. A horse was considered as habituated to the setting when it explored the setting, walked to the bucket and fed.

Each experiment included five independent phases (Table 1): baseline; unknown person; umbrella opening; ground obstacle; social isolation. These phases lasted no more than 5 min each, except for the baseline phase. Horses were led and removed from the testing area between each phase by an operator. During each testing phase, the horses were free in the testing area.

HRV data were collected using the Polar V800 Equine^®^ system (Kempele, Finland). The data collected with the Polar system were transformed with Kubios^®^ software (Kuopio, Finland) and only normal R-R intervals were considered for the HRV analysis. The final data included time domain and frequency domain analysis results (Table 2). Frequency band thresholds were established within each frequency interval using the following parameters: LF power = 0.005–0.07 Hz; HF power = 0.07–0.5 Hz; and LF/HF ratio [32].

During the last four phases, behavioral data were collected via video recording using a Sony Handycam HDR-CX450^®^ camera (Weybridge, UK). Behaviors were assessed through video analysis, and with the help of an Excel^®^ matrix (Redmond, WA, USA), behavior durations were determined by one operator according to an ethogram (Table 3). This ethogram was developed based on the importance of these behaviors for equines [18,19,38,39,40]. For experiment 1, raw data were used for statistical analysis, and the test was considered to last 5 min for each phase for each horse. However, most of the horses did not need 5 min to achieve the goal, so in the second experiment, each phase was considered as finished once the horse achieved the goal. As the time to achieve the goal could vary between individuals, the proportion of the duration of each specific behavior over the latency to obtain the treat was estimated.

### 2.3. Statistical Analysis

Statistical analysis was carried out using SAS 9.4 software (SAS Institute Inc., Cary, NC, USA). The significance threshold was classically fixed at 5%.

The behavioral parameter “interaction with another horse” was not considered in phase 5 because the untested horse was removed from the testing area.

#### 2.3.1. Experiment 1

For HR, SDNN, RMSSD, LF/HF ratio and Latency 1, the assumption of normality of the data was tested using the residual diagnostics plots and the UNIVARIATE procedure. Comparisons between phases were performed with the general linear mixed model using the MIXED procedure. If significant differences were found, multiple comparisons were analyzed with the Tukey test using the LSMEANS statement.

For Exploration and Interaction, the assumption of normality of the data was tested with the same procedure, although it was not verified. Then, comparisons between phases were performed using the nonparametric Friedman test using the FREQ procedure. If significant differences were found, multiple comparisons were analyzed with the signed-rank test for each pair of times with the UNIVARIATE procedure, and then Bonferroni correction was applied with the MULTTEST procedure to control the error risk due to the multiplicity of the tests.

#### 2.3.2. Experiment 2

For SDNN, RMSSD, LF/HF ratio and Latency 1, the assumption of normality of the data was tested using residual diagnostics plots and the UNIVARIATE procedure. Homoscedasticity was tested with Levene’s test using the GLM procedure. Comparisons between phases and activity were performed with the general linear mixed model using the MIXED procedure. When significant differences were found, multiple comparisons were analyzed with the Tukey test using the LSMEANS statement.

For HR and Exploration, the assumption of normality was verified, but the homogeneity of the variances was not. The GROUP = option was added in the REPEATED statement of the MIXED procedure. Then, comparisons between phases and activity were performed with the general linear mixed model using the MIXED procedure. If significant differences were found, multiple comparisons were analyzed with the Tukey test using the LSMEANS statement.

For Latency 2 (after the opening of the umbrella), the assumption of normality was not verified. Consequently, the nonparametric Kruskal-Wallis test was performed using the NPAR1WAY procedure. If significant differences were found, multiple comparisons were analyzed with the Wilcoxon test using the NPAR1WAY procedure, and then Bonferroni correction was applied with the MULTTEST procedure to control the error risk due to the multiplicity of the tests.

For Interaction, data were transformed into binary variables because the animals did not perform this behavior very often. In this case, the following code was considered: 1—there was an interaction with the untested horse; 0—there was no interaction with the untested horse. Comparisons between phases and activity were performed with the generalized linear mixed model using the GLIMMIX procedure. If significant differences were found, multiple comparisons were analyzed with the Tukey test using the LSMEANS statement.

## 3. Results

### 3.1. Experiment 1

Due to technical issues concerning the HR monitor, the data of 3 horses were not recorded. Additionally, artifacts concerning SDNN, RMSSD and the LF/HF ratio parameter of 2 horses were found. Aberrant data were excluded from the statistical analysis.

Additionally, for technical reasons, the video camera did not record some time frames, and some data were absent for 1 horse and could not be considered in the statistical analysis.

Some horses in the original population (one horse in phase 2; one horse in phase 3; three horses in phase 4; and two horses in phase 5) did not achieve the goal within 5 min, so their data were removed from the statistical analysis considering Latency 1.

All the included horses succeeded in the habituation phase.

#### 3.1.1. Physiological Data (Table 4)

Significant differences in HR were observed between phases (DF = 4; F = 20.55; *p* < 0.0001). More specifically, HR was significantly lower in phase 1 than in all the other phases, and HR was significantly higher in phase 3 than in phases 2 and 4 (Table 4).

There was no significant difference in SDNN between phases (DF = 4; F = 1.74; *p* = 0.1446) (Table 4).

Significant differences in RMSSD were observed between phases (DF = 4; F = 2.74; *p* = 0.0309). However, after performing the Tukey adjustment for multiple comparisons, significant differences between individual phases could not be identified (Table 4). 

There was no significant difference in the LF/HF ratio between phases (DF = 4; F = 0.45; *p* = 0.7723) (Table 4).

**Table 4:** Physiological data results of experiment 1.

Parameter	Phase	N	Mean	Std. Dev.	Median	Minimum	Maximum
Heart Rate	Baseline	41	42 ^a^	7	43	30	55
	Unknown person	39	52 ^b^	12	49	35	80
	Umbrella opening	41	58 ^bc^	13	56	34	88
	Ground obstacle	40	52 ^c^	10	50	34	78
	Social isolation	40	54	17	52	31	129
SDNN	Baseline	41	218	167	163	70	1026
	Unknown person	39	244	184	216	70	1192
	Umbrella opening	41	207	100	176	59	408
	Ground obstacle	39	177	111	145	44	482
	Social isolation	38	227	191	167	29	1024
RMSSD	Baseline	41	191	150	127	48	568
	Unknown person	39	172	103	183	29	441
	Umbrella opening	41	133	92	116	28	404
	Ground obstacle	39	124	86	90	27	409
	Social isolation	38	156	116	136	23	453
LF/HF Ratio	Baseline	41	3	3	3	0	13
	Unknown person	39	7	11	3	0	59
	Umbrella opening	41	8	13	5	0	78
	Ground obstacle	40	5	7	3	0	35
	Social isolation	40	8	15	3	0	67

^a^ Significant difference between the marked phase and all the other phases (Tukey test). ^b,c^ Significant difference between the marked phases (Tukey test).

#### 3.1.2. Behavioral Data (Table 5)

For Exploration, significant differences were observed between phases (DF = 3; Chi-square = 27.35; *p* < 0.0001). More specifically, Exploration was significantly lower in phase 5 than all the other phases (Table 5).

There was no significant difference in interactions with another horse between phases (DF = 2; Chi-square = 2.71; *p* = 0.2576) (Table 5).

Significant differences were observed between phases considering Latency 1 (DF = 3; F = 14.53; *p* < 0.0001). More precisely, Latency 1 was significantly higher in phase 2 than in phase 3. Additionally, Latency 1 was significantly lower in phase 5 than all the other phases (Table 5).

**Table 5:** Behavioral data results of experiment 1.

Parameter	Phase	N	Mean	Std. Dev.	Median	Minimum	Maximum
Exploration time (s)	Unknown person	41	42	48	22	0	199
	Umbrella opening	41	41	40	31	0	183
	Ground obstacle	40	35	53	14	0	233
	Social isolation	40	12	16	7 ^b^	0	86
Interaction time (s)	Unknown person	41	7	19	0	0	98
	Umbrella opening	41	5	12	0	0	55
	Ground obstacle	40	3	8	0	0	40
Latency 1 time (s)	Unknown person	40	71 ^c^	76	38	10	300
	Umbrella opening	40	37 ^c^	27	29	9	123
	Ground obstacle	38	48	62	24	10	300
	Social isolation	39	21 ^a^	18	14	5	89

^a^ Significant difference between the marked phase and all the other phases (Tukey test). ^b^ Significant difference between the marked phase and all the other phases (Signed-rank test). ^c^ Significant difference between the marked phases (Tukey test).

### 3.2. Experiment 2

Due to technical issues concerning the HR monitor, the data of 5 horses were not recorded. Additionally, artifacts concerning HR, SDNN, RMSSD, and the LF/HF ratio of a few horses were found. Aberrant data were excluded from the statistical analysis.

Furthermore, because of technical reasons, the video camera did not record some time frames of 1 horse, so these data were absent and could not be considered in the statistical analysis.

Some horses in the original population did not achieve the goal within the 5 min, so their data were removed from the statistical analysis for Latency 1 (one horse in phase 2, one horse in phase 3, three horses in phase 4, and two horses in phase 5) and Latency 2 (three horses only in phase 3).

All the included horses succeeded in the habituation phase.

#### 3.2.1. Physiological Data (Table 6)

There was no significant difference in HR between activities (DF = 3; F = 1.02; *p* = 0.3886) (Table 6).

Significant differences in SDNN were observed between activities (DF = 3; F = 3.36; *p* = 0.0202). More specifically, SDNN was significantly higher in the dressage group than in the EAA/T group. A trend was also observed between the jumping and the EAA/T groups, with a higher SDNN value in the jumping group than in the EAA/T group. No significant difference was observed in the phase effect or in the interaction between the activity and phase factors (Table 6).

Significant differences in RMSSD were observed between activities (DF = 3; F = 4.09; *p* = 0.0078). More specifically, RMSSD was significantly higher in the dressage group than in the EAA/T group. A trend was also observed between the jumping and the EAA/T groups, with a higher RMSSD value in the jumping group than in the EAA/T group. No significant difference was observed in the phase effect or in the interaction between the activity and phase factors (Table 6).

Significant differences in LF/HF were observed between activities (DF = 3; F = 4.79; *p* = 0.0031). More specifically, LF/HF was significantly higher in the EAA/T and eventing groups than in the dressage group. No significant difference was observed in the phase effect or in the interaction between the activity and phase factors (Table 6).

**Table 6:** Physiological data results of experiment 2.

Parameter	Activity	N	Mean	Std. Dev.	Median	Minimum	Maximum
Heart Rate	Jumping	49	50	10	49	32	81
	Eventing	65	51	13	49	30	82
	Dressage	37	50	15	49	30	85
	EAA/T	43	47	8	46	35	76
SDNN	Jumping	48	189	93	170	67	443
	Eventing	64	188	112	153	29	515
	Dressage	38	216 ^a^	114	188	57	500
	EAA/T	43	152 ^a^	94	110	32	406
RMSSD	Jumping	48	138	84	117	45	351
	Eventing	65	136	105	106	25	441
	Dressage	36	171 ^a^	106	154	23	436
	EAA/T	43	108 ^a^	89	81	32	466
LF/HF Ratio	Jumping	49	5	6	3	0	38
	Eventing	64	6 ^a^	8	4	0	54
	Dressage	38	2 ^ab^	2	2	0	12
	EAA/T	43	7 ^b^	9	4	0	57

^a,b^ significant differences between the marked phases (Tukey test).

#### 3.2.2. Behavioral Data (Table 7)

Significant differences in Exploration were observed between activities (DF = 3; F = 5.79; *p* = 0.0013). More specifically, Exploration was significantly higher in the jumping and EAA/T groups than in the eventing and dressage groups. No significant difference was observed in the phase effect or in the interaction between the activity and phase factors (Table 7).

There was no significant difference in Interaction between activities (DF = 3; F = 1.11; *p* = 0.3486). Interaction was only performed by 47% of the horses in the jumping group, 24% in the eventing group, 15% in the dressage group and 7% in the EAA/T group (Descriptive statistics: 0 = absence of interaction; 1 = presence of interaction; jumping: 0 = 16, 1 = 14; eventing: 0 = 29, 1 = 9; dressage: 0 = 23, 1 = 4; EAA/T: 0 = 25, 1 = 2) (Table 7).

Significant differences in Latency 1 were observed between activities (DF = 3; F = 8.97; *p* < 0.0001). More specifically, Latency 1 was significantly higher in the jumping and EAA/T groups than in the eventing group. Additionally, Latency 1 was significantly higher in the EAA/T group than in the dressage group. A significant difference was also observed in the phase effect (DF = 3; F = 5.87; *p* < 0.001). More precisely, Latency 1 was significantly higher in phase 2 than in phase 5. No significant difference was observed in interaction between activity and phase factors (Table 7).

There was no significant difference in Latency 2 between activities (DF = 3; Chi-Square = 4.58; *p* = 0.2052) (Table 7).

**Table 7:** Behavioral data results of experiment 2.

Parameter	Activity	N	Mean	Std. Dev.	Median	Minimum	Maximum
Exploration	Jumping	40	68 ^ab^	20	72	13	97
	Eventing	51	51 ^ac^	25	55	0	100
	Dressage	35	57 ^bd^	21	60	8	86
	EAA/T	36	69 ^cd^	29	68	14	100
Latency 1	Jumping	38	66 ^a^	69	40	10	300
	Eventing	49	29 ^ab^	31	19	8	179
	Dressage	36	44 ^c^	61	25	5	300
	EAA/T	35	82 ^bc^	77	62	7	294
Latency 2	Jumping	10	41	32	38	2	80
	Eventing	12	36	82	9	0	295
	Dressage	9	44	80	6	2	247
	EAA/T	7	7	7	6	0	18

^a,b,c,d^ significant differences between the marked phases (Tukey test).

## 4. Discussion

In general, the results obtained in this study have shown that equine behavioral and physiological responses can be influenced by the activities in which horses are involved.

The first experiment confirmed that the stimuli in the different phases were neither too frightening nor challenging for the animals. Significant differences in RMSSD meant that the parasympathetic nervous system response differed in the different phases, but an overall balance of the ANS was maintained, as demonstrated by the absence of significant differences in SDNN and the LF/HF ratio.

In terms of the HR, an increase between the baseline HR and the HR of all the other phases was expected because the baseline represents an inactive phase from both physical and psychological perspectives. In the other phases, horses were exposed to new environmental conditions that they had to explore and were thus active. Significant differences concerning HR in phase 3 were most likely associated with the physical reaction of moving away from the sudden stimulus (the umbrella opening) rather than with emotional unbalance, as no other modifications in physiological parameters were found.

The decrease in the time spent exploring the testing area, as well as the decrease in Latency 1 between phases 2 and 3, could be explained by the learning capacities of the horses. The decrease in Exploration and Latency 1 in phase 5 might be interpreted as a strategy to cope with social isolation. This coping strategy was successful in balancing the emotional state of the horses, as demonstrated in the HR, SDNN and LF/HF ratio results.

The general lack of interactions between horses was an interesting finding, especially since horses are a social species. Nevertheless, even though interactions between horses were not observed as often as expected, the removal of the familiar horse modified the behavior of the tested horse. This behavioral modification was expressed by a reduction in exploratory behavior and Latency 1. These findings mean that even if the horses did not interact, the presence of a familiar horse appeared to have an emotional value for the tested horse.

To investigate the influence of equines’ activities on the emotional responses of horses, the selected stimuli should not create excessive fear. This condition was important because stimuli that are too frightening may induce maladaptive responses [41,42], which was not the subject of the present investigation. The first experiment demonstrated that the stimuli were suitable for the investigation of the influence of equines’ activities on horses’ emotional responses.

The results of the second experiment showed that the activities in which horses are involved influence horses’ physiological and behavioral responses to different stimuli.

Jumping horses balanced the activity of the ANS during the test, as demonstrated by the SDNN and LF/HF ratio results. This group required more time than the dressage and eventing groups to explore the testing area before approaching the person and obtaining the reward, as expressed by the Exploration and Latency 1 results. Nevertheless, the time spent exploring did not induce any modification of ANS activity.

Dressage horses had a higher value of SDNN than EAA/T horses, meaning that the former experienced a higher overall variability of the ANS than the latter during the test. However, the differences in the RMSSD and LF/HF ratio results demonstrated a higher influence of the parasympathetic nervous system in the overall variability of horses in the dressage group than in the EAA/T group. The EAA/T group had a lower overall variability and higher sympathetic nervous system activity than the dressage group during the test, as described by the RMSSD and LF/HF ratio results. This means that horses in the dressage group were more successful than horses in the EAA/T group in balancing the ANS during the test. Dressage horses took less time than EAA/T horses to explore the testing area and approach the person and the bucket to obtain the treat. Considering the physiological data, EAA/T horses showed an unbalance in the ANS and needed more time than dressage horses to approach the unknown person, which suggests that some social apprehension in horses may be induced by equine-assisted activities/therapy.

Eventing horses also had a high LF/HF ratio. Even if no other differences were found considering the physiological data, these results mean that an unbalance in the ANS was induced by the test, and this unbalance was most likely related to the sympathetic nervous system, as no differences were found in RMSSD. Eventing horses approached the person and achieved the goal more quickly than jumping horses, but eventing horses experienced an emotional unbalance, while jumping horses did not.

All horses needed a similar length of time to approach the person after the opening of the umbrella. This is an interesting finding because it means that, independent of the activity they have been trained to practice, horses will demonstrate the same response to a sudden stimulus: running away and then returning and exploring the area.

The influence of the equine’s activity on equine behavioral and physiological responses has been studied by different authors [8,9,10,17,43].

Some authors have suggested that jumping horses react significantly less than other horses to novelty [9]. Indeed, in our test, the jumping horses had a lower reactivity than the other horses. This low reactivity is usually desired in jumping horses by riders to reduce the risk of accidents [9]. However, some undesirable side effects can be expected, such as undesirable behaviors related to low fearfulness [9].

Another study [8] described differences in the reactional state of horses from different activities, suggesting that dressage horses could be more emotionally reactive than other horses. In agreement with the cited studies, the dressage horses reacted quickly to the different stimuli in our study. However, they rapidly recovered emotional homeostasis and were more successful in coping with the different stimuli than eventing and EAA/T horses. These findings suggest that dressage horses react to different stimuli faster than the other horses, but they also recover faster than other horses from an emotional challenge. A factor that could have a role in the observed difference between the reactivity of dressage horses in different studies is the housing conditions of the animals and their temperament [43,44,45]. Horses living in single boxes are deprived of many stimuli and may become more reactive to novelty, while horses with access to paddocks, likes the ones involved in this study, may be less reactive [22,23]. Additionally, equitation science has encouraged the application of training techniques that improve equine welfare in recent years [46], which may result in horses that are less inhibited and learn to manage their emotions in different situations [47].

A difference in the endocrinal responses (salivary cortisol) between eventing and dressage horses was previously described [10]. In the present study, eventing horses showed a higher increase in sympathetic system activity than jumping and dressage horses. These differences suggest that either the endocrinal or autonomic responses of eventing horses differ from those of other horses.

Studies on EAA/T horses have suggested that therapeutic riding may be as stressful as recreational riding [15,17]. In our study, even free horses demonstrated some emotional responses (HRV and behavior results) and probably some apprehension in approaching the person. These findings suggest that EAA/T horses may cope well with therapeutic activities but have some apprehension towards human contact; thus, producing poorer interspecific socialization.

The training exercises and conditions for performing different activities are very variable as horses need to acquire different competences to succeed in each of the activities, as described previously in [12,16,17]. The different learning situations associated to specific training for the different activities may influence the behavioral and physiological responses of horses in adulthood.

Equine welfare can be influenced by handling, training and housing conditions [44,48,49,50]. This investigation highlighted the variability of equine behavioral and physiological responses to different stimuli, and the influence of some equine activities on the reactional state and emotional management of adult horses. This means that the specific training for different activities may influence equine welfare in different ways. Understandings of equine perception of different stimuli and the corresponding emotional responses are of great importance to the equestrian community and for improving equine welfare. 

## 5. Conclusions

The activity in which adult horses are involved appears to have an influence on their behavioral and physiological responses to different stimuli, and thus affects the welfare of horses. Understanding this influence is of great interest for improving equine welfare, with the aim of habituating and/or desensitizing horses to disturbing stimuli.

It might be interesting to study the same responses in foals before starting the training process for any of the described activities. This investigation would allow us to better understand how the genetic component, and the learning process through training and environmental stimulation are related in the development of behavioral and physiological responses of horses.

Further studies should be performed to acquire more knowledge about equine behavior and physiological responses related to emotions. Equine housing and general management conditions have been improved thanks to increased knowledge based on scientific evidence. Therefore, strategies to balance the emotional state of equines will probably change in the future.

## Figures and Tables

**Table 1:** Description of the aims of each phase of the experiment.

Phase	Aim	Description
Baseline (1)	Collection of HRV data at rest	HRV data was collected over a period of twenty minutes under normal life conditions of the horse (paddock or box) without any human influence.
Unknown person (2)	To test interspecific socialization	An unknown person sat on a chair close to the bucket (1 m of distance), so the horse needed to approach the person to access the treat.
Umbrella opening (3)	To test responses to a sudden stimulus	This stimulus has been used in many studies to investigate equine responses to sudden stimuli [37,38]. In this phase, the person sitting on the chair opened an umbrella when the horse starts eating from the bucket.
Ground obstacle (4)	To test recovery capacities and strategies	Obstacle bars were placed in the middle of the testing area, dividing the setting into two parts. To approach the treat and the person on the chair, the horse had to cross the bars.
Social Isolation (5)	To test equine responses to social isolation	The familiar horse and the person were removed from the testing area and from the horse’s visual field.

**Table 2:** Adapted description of HRV variables from Stucke et al., (2015) [29].

Variable	Definition	Interpretation
HR (bpm)	Heart rate mean	Overall variability of cardiac activity
SDNN (ms)	Standard deviation of RR intervals	Long-term variability of cardiac activity influenced by the autonomous nervous system
RMSSD (ms)	Square root of the mean of the sum of the squares of differences between successive interbeat-intervals (IBI)	High frequency of IBI variations correlated to parasympathetic activity
LF/HF ratio	Low frequency/high frequency ratio	Representation of the balance between sympathetic and parasympathetic nervous systems

**Table 3:** Ethogram used for video analysis.

Behavior	Definition
Exploration	The animal searches for information by walking around the testing area or by sniffing or touching the person or object.
Interactions with another horse (Interaction)	Interactions with the untested horse through physical or visual contact.
Latency to obtain the treat (Latency 1)	Time the horse takes to approach the bucket and obtain the treat in all the phases.
Latency to obtain the treat after the umbrella opens (Latency 2; only in the 2nd experiment)	Time the horse takes to approach the bucket again after the umbrella opens in phase 3.
